# Genetic Variation and Alleviation of Salinity Stress in Barley (*Hordeum vulgare* L.)

**DOI:** 10.3390/molecules23102488

**Published:** 2018-09-28

**Authors:** Mohamed A. El-Esawi, Ibrahim A. Alaraidh, Abdulaziz A. Alsahli, Hayssam M. Ali, Aisha A. Alayafi, Jacques Witczak, Margaret Ahmad

**Affiliations:** 1 Botany Department, Faculty of Science, Tanta University, Tanta 31527, Egypt; 2 Botany and Microbiology Department, College of Science, King Saud University, P.O. Box 2455, Riyadh 11451, Saudi Arabia; ialaraidh@ksu.edu.sa (I.A.A.); aalshenaifi@ksu.edu.sa (A.A.A.); hayhassan@ksu.edu.sa (H.M.A.); 3 Timber Trees Research Department, Sabahia Horticulture Research Station, Horticulture Research Institute, Agriculture Research Center, Alexandria 21526, Egypt; 4 Biological Sciences Department, Faculty of Science, University of Jeddah, Jeddah 21577, Saudi Arabia; aal_shareaf@hotmail.com; 5 UMR CNRS 8256 (B2A), IBPS, Université Paris VI, 75005 Paris, France; jacques.witczak@upmc.fr (J.W.); margaret.ahmad@upmc.fr (M.A.); 6 Department of Biology, Xavier University, Cincinnati, OH 45207, USA

**Keywords:** AFLP, European barley, genetic diversity, population structure, salt tolerance, ALA

## Abstract

Barley (*Hordeum vulgare* L.) represents one of the most important cereals cultivated worldwide. Investigating genetic variability and structure of barley is important for enhancing the crop productivity. This study aimed to investigate the diversity and structure of 40 barley genotypes originated from three European countries (France, the Netherlands, Poland) using amplified fragment length polymorphisms (AFLPs). It also aimed to study 5-aminolevulinic acid (ALA) effect on salinity tolerance of six barley genotypes. The expected heterozygosity (*H*_e_) diverged from 0.126 to 0.501, with a mean of 0.348. Polymorphic information content (*PIC*) diverged from 0.103 to 0.482 across barley genotypes, with a mean of 0.316, indicating that barley genotypes are rich in a considerable level of genetic diversity. The 40 barley genotypes were further studied based on their geographical origin (Western Europe and Eastern Europe). The Eastern European region (Poland) has a higher barley variability than the Western European region (France and the Netherlands). Nei’s distance-based cluster tree divided the 40 barley accessions into two major clusters; one cluster comprised all the varieties originated from the Eastern European region, while the other major cluster included all accessions originated from the Western European region. Structure analysis results were in a complete concordance with our cluster analysis results. Slaski 2, Damseaux and Urbanowicki genotypes have the highest diversity level, whereas Carmen, Bigo and Cambrinus genotypes have the lowest level. The response of these six varieties to NaCl stress was also investigated. Salt stress (100 mM NaCl) slightly decreased levels of chlorophyll, carotenoid and osmolytes (proteins, soluble sugars, phenolics and flavonoids) in the leaves of Slaski 2, Damseaux and Urbanowicki genotypes at non-significant level, as compared to control samples. However, pigment contents and osmolytes in leaves of Carmen, Bigo and Cambrinus genotypes were significantly decreased by salt stress. Antioxidant enzyme activities were significantly increased in Slaski 2 genotype, but non-significantly increased in Carmen by salt stress. Priming Slaski 2 and Carmen cultivars with ALA under salt stress significantly induced pigment contents, antioxidants enzymes activity and stress-responsive genes expression, relative to NaCl-stressed plants. In conclusion, this study suggested a correlation between variability percentage and degree of salinity resistance. ALA improved salt tolerance in barley.

## 1. Introduction

Barley (*Hordeum vulgare* L.) belongs to the major cereals grown in various environments worldwide [1,2]. It has a high economic value and is used in food and industry. Barley is adapted to different conditions and tolerant to several abiotic factors, including temperature, salinity, and water stress [2,3]. Cultivated barley and its wild progenitor are included in the primary gene pool [2,4]. Wild type has a higher allelic diversity than that of the cultivated one [5,6]. Wild type is also rich in genes that enable it to adapt to various abiotic and biotic factors [2,7]. Characterization and exploitation of such genes increase barley crop productivity and quality and augment crop breeding and conservation strategies.

Evaluation of genetic diversity levels and abiotic stress tolerance of crops is important for developing effective breeding programs to enhance crop productivity. Various methods have been applied to study the genetic variability and physiological mechanisms in plant species [8,9,10,11,12,13,14,15,16,17,18,19,20,21,22,23,24,25,26,27,28,29]. Over the last few years, different molecular traits including amplified fragment length polymorphisms (AFLPs), microsatellites (SSRs), diversity array technology (DArT) and inter simple sequence repeats (ISSRs) have been successfully utilized to study the variability for salinity tolerance in various plant species which would help identify salt tolerant genotypes and quantitative trait loci (QTLs) controlling salt tolerance for future exploitation in breeding programs to improve the crops and develop newly high salt tolerant varieties. For example, Krishnamurthy et al. [30] have successfully evaluated 94 rice varieties for salt tolerance using SSR markers located in the chromosome 1 *Saltol* QTL conferring salinity tolerance. The importance of SSR markers was also reported in studying *Brassica* breeding for salinity tolerance and identification of QTLs conferring salt tolerance [31]. Moreover, Kumar et al. [32] identified the variation in salinity tolerance in both *Brassica* and rice using SSR markers. Fan et al. [33] identified important QTLs conferring salt and drought tolerance using physiological and agronomic traits in barley population having a linkage map constructed based on several SSR, AFLP and DArT markers. Ahmadi-Ochtapeh et al. [34] also identified and characterized promising QTLs controlling salinity tolerance in barley population having a linkage genetic map constructed based on 106 SSR and AFLP markers.

Molecular makers have also been successfully used in detecting genetic variation levels and phylogenetic relationships in various plant species [35,36,37]. Moreover, genetic diversity and relationships have also been studied in barley germplasm collected from different geographical origins using morphological, biochemical, and molecular traits [2,38,39]. SDS-PAGE and isozyme analyses were used for studying the genetic diversity of Brazilian barley genotypes and showed diversity levels [40]. As morphological and biochemical characters might be influenced by surrounding environment, molecular traits have been therefore used to assess polymorphism and population structure. Random amplified polymorphic DNAs (RAPDs) were also used to evaluate the variability levels in barley [41]. Furthermore, AFLP and SSR markers proved useful for assessing barley diversity levels and structure due to their high polymorphism levels and exhibiting a considerable number of amplified fragments which are useful in diversity studies [2,42]. AFLPs and microsatellites have been successfully used to evaluate barley germplasm of various origins [2,5,6,39,43,44]. Those studies recorded different levels of diversity and provided useful information for understanding the diversity and relationships of barley; however most of them have analyzed a limited range of genotypes. Therefore, more information on genetic diversity, structure, and relationships of barley genotypes of different origins is still required. Genebanks and genetic resource centers maintain barley germplasm that need further genetic characterization to exploit their genetic diversity and tolerance to abiotic stresses in breeding programs for enhancing the crop quality and yield. The objectives of the current investigation were therefore to study variability level, structure, and relationships of 40 barley genotypes originated from three European countries (France, the Netherlands, Poland) using AFLP markers. Additionally, this study aimed at investigating the correlation between levels of diversity and salinity (NaCl) tolerance in some cultivars contrasting in their genetic diversity levels, and evaluated the 5-aminolevulinic acid (ALA) growth regulator effects on salt tolerance in those cultivars.

## 2. Results and Discussion

### 2.1. Molecular Diversity Analysis in Barley Genotypes

#### 2.1.1. Polymorphism Analysis and Diversity Indices of AFLP Markers

Several methods have been applied in assessing genetic diversity levels in crops to enhance their productivity and quality. Because morphological traits are influenced by environmental conditions, molecular markers have been efficiently utilized in barely variability studies. AFLP proved to be a powerful technique for cultivar identification characterization. The current study used AFLP markers to characterize the molecular variability and structure of barley varieties originated from three countries (France, the Netherlands, and Poland). Across the 14 AFLP primer sets used, 760 AFLP bands were recorded in the 160 individuals of the 40 varieties studied (Table 1). The AFLP fragments varied in molecular size from 45 to 785 bp, and in number from 28 to 75 with an average of 54.29 (Table 1). Primer pair (E-AGG/M-CAG) revealed the lowest fragment number (28), with molecular sizes varying from 55 to 490 bp (Table 1). The primer set (E-ACC/M-CAG) revealed the highest fragment number (75), with molecular sizes varying from 75 to 780 bp. All the 14 AFLP primer sets were polymorphic. Polymorphic fragments diverged in number from 18 (E-AAG/M-CTT) to 52 (E-AGG/M-CTA) with a mean of 35.57. This mean was lower than that recorded in barley (58.5) by Assefa et al. [45], but higher than that recorded by Varshney et al. [46] for barley (27). Furthermore, the polymorphism percentage varied from 45.33% (E-ACC/M-CAG) to 92.73% (E-ACT/M-CAG), with an average of 66.07% (Table 1). This mean polymorphism percentage was relatively similar to that reported by Assefa et al. [45], but higher than that recorded by Adawy et al. [44]. The gene diversity of AFLP primer sets diverged from 0.17 (E-AAG/M-CTG) to 0.48 (E-ACT/M-CAG), with an average of 0.37, indicating a good level of genetic diversity within barely genotypes. Polymorphic information content (*PIC*) exhibited a mean of 0.33 (Table 1). This mean was higher than that indicated by Varshney et al. [46]. The difference in this data is a result of the differences in the barely genotypes analyzed, and molecular markers used. The AFLP primer sets revealing a high degree of polymorphism in the present study are recommended for use in future diversity studies of barley germplasm.

#### 2.1.2. Genetic Diversity of Barley Genotypes

Table 2 exhibits the genetic variability indices calculated for each of the 40 barley genotypes. Observed heterozygosity (*H*_o_) diverged from 0.127 (variety Chevalier from France) to 0.374 (Slaski 2 from Poland). Expected heterozygosity (*H*_e_) diverged from 0.126 (Carmen from France) to 0.501 (Slaski 2 from Poland), with a mean of 0.348. *PIC* also diverged from 0.103 (Carmen from France) to 0.482 (Slaski 2 from Poland), with a mean of 0.316. These results indicate that Carmen cultivar comprised the lowest diversity level, whereas Slaski 2 comprised the highest diversity level. Three accessions (Damseaux from France, Urbanowicki from Poland, and Slaski 2 from Poland) exhibited negative fixation indices (*F*) (Table 2), indicating the presence of higher level of heterozygosity in those accessions.

Moreover, the 40 barley genotypes studied were split into 2 groups based on their geographical location (Western Europe and Eastern Europe). Western Europe includes the accessions originated from France and the Netherlands, while Eastern Europe includes the accessions originated from Poland. Table 3 shows the diversity indices calculated for the barley genotypes of Western and Eastern European regions. The observed heterozygosity (*H*_o_) of the Western and Eastern European regions was 0.247 and 0.267, respectively. The expected heterozygosity (*H*_e_) of the Western and Eastern European regions was 0.335 and 0.380, respectively. The fixation index of the Western and Eastern European regions was 0.261 and 0.327, respectively. *PIC* value of the Western and Eastern European regions was 0.298 and 0.368, respectively. These diversity indices results indicate that the Eastern European region (Poland) has a higher barley variability than the Western European region (France and Netherlands). Therefore, barley genotypes originated from Poland could be further exploited to improve the crop.

#### 2.1.3. Cluster Analysis and Population Structure of Barley Genotypes

**Figure 1:**
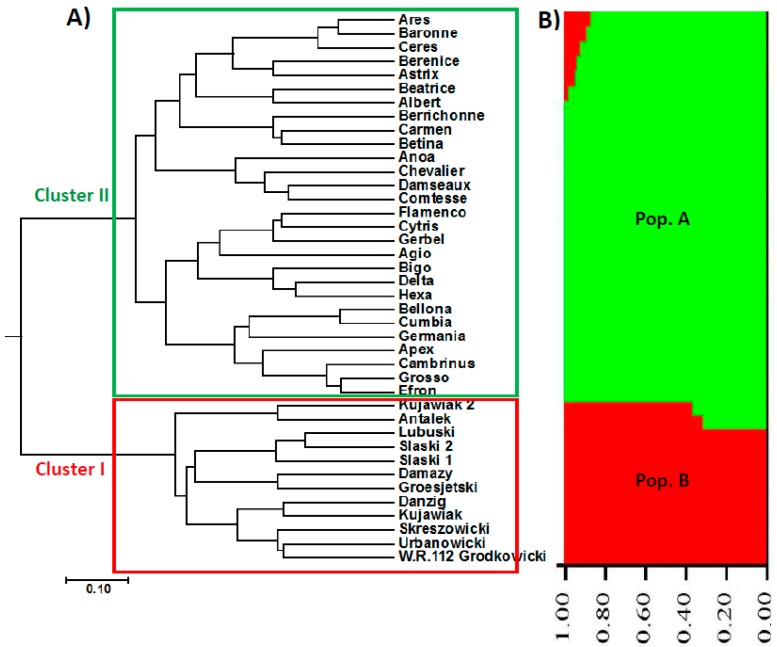
UPGMA phylogenetic tree and population structure (at *K* = 2) of the 40 barley accessions based on AFLP data; (**A**) Cluster I included the Eastern European genotypes (Poland), while Cluster II represents the Western European genotypes (France and the Netherlands). (**B**) Structure analysis showing that the 40 barely accessions are assigned to two populations (Pop. A represented in green comprised the Western European genotypes, while Pop. B represented in red included the Eastern European genotypes).

Nei’s distance-based cluster tree was performed using AFLP data and exhibited the relationships among the 40 barley accessions studied (Figure 1). The dendrogram split into 2 major clusters; one cluster had all the 12 barley accessions originated from the Eastern European region (Poland), while the other cluster included all the barley accessions originated from the Western European region (France and the Netherlands) and split into two sub-clusters. The first one included 11 out of the 12 accessions originated from the Netherlands (Agio, Bigo, Delta, Hexa, Bellona, Cumbia, Germania, Apex, Cambrinus, Grosso, and Efron) and three accessions from France (Flamenco, Cytris, Gerbel). The second sub-cluster comprised one accession originated from the Netherlands (Anoa) and the remaining 13 accessions originated from France (Damseaux, Comtesse, Chevalier, Ceres, Carmen, Betina, Berrichonne, Berenice, Beatrice, Baronne, Astrix, Ares, Albert). The cluster analysis results indicate that barley accessions originated from the Western European region (France and the Netherlands) were more closely related to each other than to that originated from Eastern Europe (Poland).

**Figure 2:**
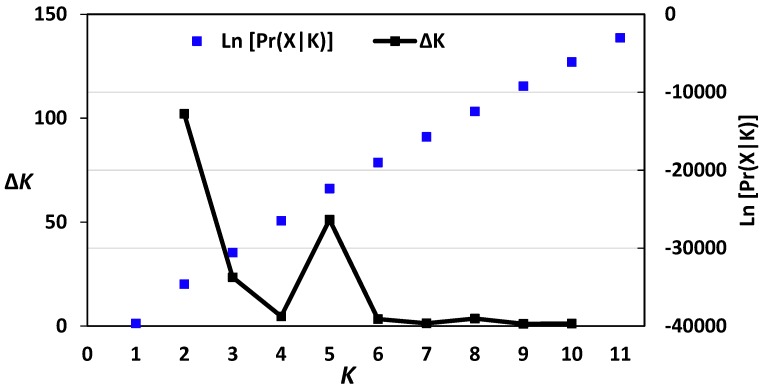
Log-likelihood and Δ*K* values of structure analysis of the 40 barley accessions based on AFLP data. *K* = 2 had the highest Δ*K* value, indicating that the 40 barely accessions could be assigned to two populations.

Genetic structure of the 40 barley accessions was studied using the software STRUCTURE 2.3. This genetic analysis can estimate the hypothetical populations number to which barley accessions should be assigned. Structure analysis of barely genotypes revealed that *K*= 2 had the highest Δ*K* value (Figure 2). Additionally, the fourth run was the best among the ten runs for *K*= 2 based on the likelihood values. Consequently, *K*= 2 represented the genetic structure of barely accessions studied, indicating that the 40 barely accessions could be assigned to two populations. At *K* = 2, all barely genotypes originated from Western Europe (16 genotypes from France and 12 genotypes from the Netherlands) were assigned to one population (Pop. A) (Figure 1), while all genotypes from the Eastern European region (12 genotypes from Poland) were included the second population (Pop. B). The genetic structure results were in harmony with our cluster analysis data, confirming that barley accessions originated from Western Europe (France and the Netherlands) were more closely related to each other than to that originated from Eastern Europe (Poland).

### 2.2. 5-Aminolevulinic Acid Effect on Salt Tolerance in Barley

Our AFLP results showed that Slaski 2, Damseaux and Urbanowicki genotypes contained the highest genetic diversity level, whereas Carmen, Bigo and Cambrinus genotypes comprised the lowest diversity level (Table 2). We, Therefore, chose these six variable genotypes to study their responsiveness to salt stress and evaluate whether correlation between their variability percentage and salinity tolerance level exists. Effects of 5-aminolevulinic acid on the tolerance of those six genotypes to salt stress were also studied.

The salt stress (100 mM NaCl) slightly decreased levels of chlorophyll, carotenoids and osmolytes (proteins, soluble sugars, phenolics and flavonoids) in the leaves of Slaski 2, Damseaux and Urbanowicki genotypes at non-significant level, as compared to controls (Table 4, Table 5 and Table 6). Moreover, salt stress slightly increased malondialdehyde (MDA) and hydrogen peroxide levels in leaves of Slaski 2, Damseaux and Urbanowicki genotypes. Treating the salt-stressed Slaski 2, Damseaux and Urbanowicki genotypes with ALA significantly induced the biosynthesis of pigments and osmolytes but significantly reduced the levels of hydrogen peroxide and MDA, relative to the plants treated with NaCl alone (Table 4, Table 5 and Table 6). Furthermore, priming the non-stressed Slaski 2, Damseaux and Urbanowicki genotypes with ALA significantly augmented the synthesis of pigments and osmolytes but reduced the levels of hydrogen peroxide and MDA, as compared to the untreated control plants. ALA enhanced proline accumulation and the antioxidant activity (DPPH) in the three salt-stressed genotypes (Table 4, Table 5 and Table 6).

On the other hand, salt stress (100 mM NaCl) significantly decreased the contents of photosynthetic pigments and osmolytes in the leaves of Carmen, Bigo and Cambrinus genotypes, as compared to the untreated control plants (Table 7, Table 8 and Table 9). Moreover, NaCl stress significantly enhanced hydrogen peroxide and MDA contents in leaves of Carmen, Bigo and Cambrinus genotypes. Treating the salt-stressed Carmen, Bigo and Cambrinus genotypes with ALA significantly induced pigments biosynthesis and osmolytes contents but significantly reduced the levels of hydrogen peroxide and MDA, relative to the plants treated with salt alone (Table 7, Table 8 and Table 9). Furthermore, priming the control Carmen, Bigo and Cambrinus plants with ALA significantly induced photosynthetic pigments biosynthesis and osmolytes contents but reduced the levels of hydrogen peroxide and malondialdehyde, as compared to the untreated control plants (Table 7, Table 8 and Table 9). Moreover, ALA treatment enhanced proline biosynthesis and antioxidant activity (DPPH) in those three salinity-stressed barley genotypes (Table 7, Table 8 and Table 9).

**Figure 3:**
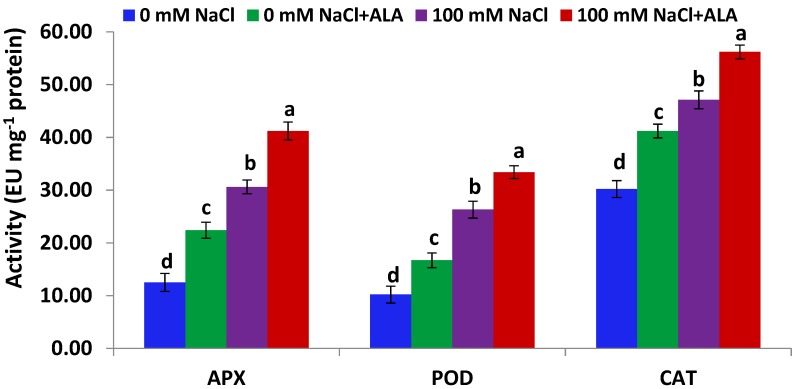
APX, POD and CAT activities in Slaski 2 cultivar leaves under salt and/or ALA (7 ppm) effects. Values represent means ± SE (*n* = 4). Different letters show significant difference among treatments (*p* ≤ 0.05).

**Figure 4:**
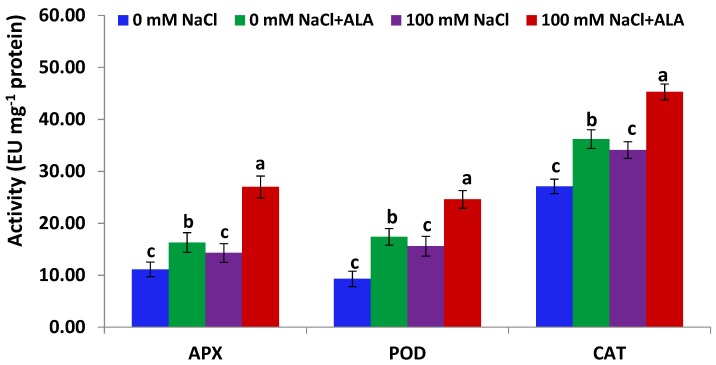
APX, POD and CAT activities in Carmen cultivar leaves under salt and/or ALA (7 ppm) effects. Values represent means ± SE (*n* = 4). Different letters show significant difference among treatments (*p* ≤ 0.05).

Figure 3 indicates that antioxidant enzyme (APX, POD and CAT) activities in Slaski 2 leaves were significantly activated under salinity stress. Additionally, treating Slaski 2 genotype with ALA under salt stress significantly augmented enzyme activities, compared to plants dealt only with salt (Figure 3). Furthermore, ALA significantly promoted antioxidant enzyme activities in non-stressed Slaski 2 plants, relative to control plants. On the other hand, Figure 4 indicates that Carmen leaves subjected to NaCl stress exhibited non-significantly induced levels of antioxidant enzyme activities, as compared to control samples. Such an increase was less than that reported in Slaski 2 variety. ALA significantly augmented the activities of antioxidant enzymes in Carmen genotype subjected to salt stress, relative to NaCl-stressed plants (Figure 4). Furthermore, ALA significantly augmented antioxidant enzyme activities in non-stressed Carmen plants, as compared to control plants.

**Figure 5:**
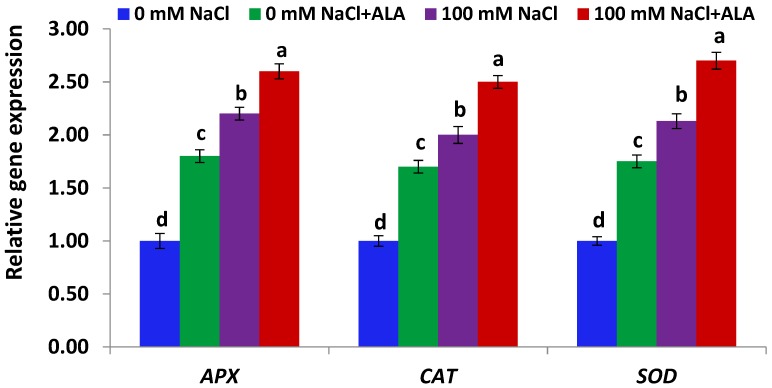
Expression level of antioxidant genes in Slaski 2 cultivar leaves under salt and/or ALA (7 ppm) effects. Values represent means ± SE (*n* = 4). Different letters show significant difference among treatments (*p* ≤ 0.05).

**Figure 6:**
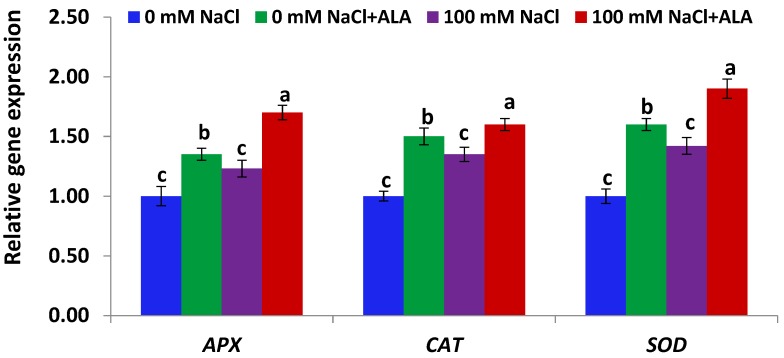
Expression level of antioxidant genes in Carmen cultivar leaves under salt and/or ALA (7 ppm) effects. Values represent means ± SE (*n* = 4). Different letters show significant difference among treatments (*p* ≤ 0.05).

Figure 5 indicates antioxidant genes (*APX*, *CAT* and *SOD*) expression in Slaski 2 leaves was significantly up-regulated under high salinity conditions, as compared to control plants. ALA significantly enhanced antioxidant genes expression in Slaski 2 plants subjected to NaCl stress, relative to NaCl-treated plants (Figure 5). Moreover, ALA treatment significantly augmented antioxidant genes expression in non-stressed Slaski 2, relative to control plants. Figure 6 indicates that antioxidant genes expression in Carmen leaves was non-significantly enhanced under NaCl conditions, relative to control plants. Such an increase was less than that reported in Slaski 2 leaves. ALA significantly augmented the antioxidant genes expression in the salt-stressed Carmen plants, as compared to NaCl-treated plants (Figure 6). Moreover, ALA significantly enhanced the antioxidant genes expression in the non-stressed Carmen plants, as compared to control plants.

The above results showed Slaski 2, Damseaux and Urbanowicki genotypes tolerated salt stress more than Carmen, Bigo and Cambrinus genotypes. This result suggests that Slaski 2, Damseaux and Urbanowicki genotypes (having the highest diversity level) are moderately tolerant to salt stress, whereas Carmen, Bigo and Cambrinus genotypes (having the lowest diversity level) are sensitive to salt stress, suggesting sort of correlation between level of variability and degree of salinity tolerance. Moreover, results showed that Slaski 2, Damseaux and Urbanowicki plants enhanced their self-defense mechanisms by up-regulating their antioxidant enzymes under high salinity stress to alleviate the resulting oxidative damage. Results also showed a key role of ALA to further improve salinity tolerance in those six barley cultivars by up-regulating photosynthetic pigment biosynthetic pathway and expression of antioxidant genes. ALA has played key roles against the abiotic stresses-induced inhibitory in various plants. ALA Foliar spray alleviated the adverse effects of high salinity in creeping bentgrass [47]. Exogenous ALA also augmented salinity tolerance in rice [48], tomato [49], sicklepod [50], swiss chard [51], and cucumber [52]. Additionally, exogenous ALA boosted chlorophyll content in lettuce subjected to UV-B stress [53]. ALA also increased chlorophyll fluorescence indices in rapes exposed to drought conditions [54]. Furthermore, ALA also induced expression of genes mediating photosynthesis Calvin cycle in rapes exposed to drought stress [55].

In conclusion, the current study reported a considerable AFLP-based genetic diversity level in the barley germplasm studied and might be exploited to improve crop yield and quality. Results also indicated a correlation between level of variability and degree of salinity tolerance in barley cultivars. ALA improved salt tolerance in barley cultivars.

## 3. Materials and Methods

### 3.1. Plant Material

Forty European barley accessions were brought from the Center for Genetic Resources in Netherlands (Table 2). Those varieties included 16 genotypes originated from France, 12 genotypes from the Netherlands, and 12 genotypes from Poland.

### 3.2. Molecular Diversity Analysis

#### 3.2.1. DNA Isolation and AFLP Analysis

Total DNA was prepared from the 4-week old leaf tissues of each genotype by DNeasy Plant Mini Kit (Qiagen, Hilden, Germany). Five DNA samples were isolated from each of the 40 genotypes and used for AFLP analyses. AFLP analyses were done as reported by Vos et al. [56] with little changes as shown in Adawy et al. [44]. Briefly, DNA samples were digested with *MseI* and *EcoRI* for 2 h at 37 °C, then left at 70 °C for 15 min., then ligated to MseI and EcoRI adapters for 4 h at 20 °C. Preamplification was conducted in a DNA thermocycler (Applied Biosystems, Foster City, CA, USA) for 20 cycles (94 °C/30s, 56 °C/60 s and 72 °C/60 s). Amplified products were then diluted and used for selective amplification. Selective amplification reactions were done using 14 primer pairs (Eurogentec SA) reported by Adawy et al. [44] in a DNA thermocycler set up as follows: one cycle at 94 °C/30 s, 65 °C/30 s and 72 °C/60 s, followed by 13 cycles during which the annealing temperature decreased by 0.7 °C every cycle. This was then followed by 23 cycles at 94 °C/30 s, 56 °C/30 s and 72 °C/60 s. The 14 primer sets comprised E-AAC/M- CTT, E-ACT/M-CTG, E-ACA/M-CAG, E-ACT/M-CAG, E-AGG/M-CTA, E-AAG/M-CTT, E-ACT/M-CTT, E-ACC/M-CTA, E-AGG/M-CAG, E-AAG/M-CTG, E-AGG/M-CAC, E-ACC/M-CAC, E-AGG/M-CTT, and E-AGG/M-CAG (Table 1). Polyacrylamide gels (8%, *w*/*v*) were used to analyze amplified PCR products. SYBR Gold stain was used to stain gels.

#### 3.2.2. Data Analysis

AFLP fragments were represented as present (1) and absent (0), forming binary data sets. PowerMarker 3.25 [57] and GenAlEx 6.5 [58] were used to estimate variability indices such as polymorphism, observed heterozygosity (*H_o_*), fixation index (*F*), polymorphic information content (*PIC*), gene diversity, and expected heterozygosity (*H_e_*). UPGMA cluster analysis was carried out based on Nei’s genetic distance [59].

The genetic structure of the 40 barley genotypes was studied using STRUCTURE 2.3 software [60]. The analyses were done using hypothetical populations numbers (*K*) ranging from 1 to 11, along with 100,000 burn-in run iteration as well as 100,000 Markov chain Monte Carlo, and with ten runs for each *K* value. Structure Harvester [61] was applied and showed the best likely values of *K* [62].

### 3.3. Salt Tolerance Test and 5-Aminolevulinic Acid Effects on Barley

#### 3.3.1. Growth Condition and Treatments

Barley cultivars (Slaski 2, Damseaux, Urbanowicki, Carmen, Bigo and Cambrinus) seeds were sterilized for 4 min in 5% NaClO solution, washed five times in distilled H_2_O, and left to grow in a growth chamber for 5 days. Barley seedlings were then transferred into plastic pots (one seedling per pot, five replicates) having soil comprising perlite, sand, and peat (1:1:1). Randomized pots were kept under conditions of 16/8 h, 26/21 °C, and 80% humidity. After transplantation immediately, plants were watered daily using Hoagland nutrient containing 0 and 100 mM NaCl over 3 weeks. 5-aminolevulinic acid foliar sprays (7 ppm) were applied weekly (i.e., every 7 days) to barley plants. After 3 weeks of transplantation, leaves were harvested for physiological analyses.

#### 3.3.2. Estimation of Contents of Photosynthetic Pigments

Leaf chlorophyll and carotenoid contents were measured spectrophotometrically as indicated by Lichtenthaler and Wellburn [63]. Absorbance was spectrophotometrically measured at 452.5, 663, 644, and 452.5 nm for carotenoids, chl a and chl b, respectively. 80% acetone was served as a blank.

#### 3.3.3. Estimation of Osmolytes, H_2_O_2_, MDA, and Antioxidant Activity (DPPH)

Total leaf protein content was calculated using Bradford protocol [64]. Total soluble sugar was determined as indicated by Dey [65], and absorbance was reported at 485 nm. Leaf proline content was calculated as mentioned by Bates et al. [66].

Hydrogen peroxide (H_2_O_2_) was measured as reported by Velikova et al. [67]. Malondialdehyde (MDA) was calculated as reported by Heath and Packer [68].

Total leaf phenolic content was determined as reported by Zieslin and Ben-Zaken [69]. Total leaf flavonoid content was investigated following Zhishen et al. [70]. Optical density was taken at 510 nm. Leaf antioxidant capacity was estimated using 2,2′-diphenypicrylhydrazyl (DPPH) [71]. Absorbance was taken at 517 nm.

#### 3.3.4. Antioxidant Enzyme Assay

Peroxidase (POD), ascorbate peroxidase (APX) and catalase (CAT) activities were investigated in fresh leaves using the protocol of Zhang and Kirkham [72]. Optical density was measured at 290 nm (APX) or 470 m (POD) or 240 nm (CAT).

#### 3.3.5. Transcription Analyses

Quantitative real-time PCR (qRT-PCR) analyses were conducted to evaluate expressions of 3 genes conferring salt tolerance in barley leaves. These three genes represent antioxidants genes (*APX*, *CAT* and *SOD*). Total RNA extraction was carried out from barley leaves by RNeasy Plant Mini kit (Qiagen). cDNA synthesis was made by Qiagen Reverse Transcription kit. Quantitative RT-PCR was done by Qiagen QuantiTect SYBR Green PCR kit. The conditions of PCR amplifications were: 10 min at 95 °C; 40 cycles of 20 s at 94 °C, 30 s at 60 °C, 2 min at 72 °C; and 4 min at 72 °C. Gene specific-primers were used for PCR amplification [17]. Melting-curve analyses were then applied. UBIQUITIN (*UBQ1*) was used for normalization, and the relative expression was determined by 2^−ΔΔ*C*t^ method.

#### 3.3.6. Statistical Analysis

One-way analysis of variance (ANOVA) as well as Duncan’s multiple range tests were performed. Values at *p* ≤ 0.05 differ significantly. Data represent means ± SE (*n* = 4 representing different plants). 

## Figures and Tables

**Table 1:** AFLP primers, fragments number and genetic variability indices in the 40 barely varieties.

AFLP Primer Sets	Number of Fragments	No. of Polymorphic Fragments	Polymorphism (%)	Fragments Size Range	Gene Diversity	*PIC*
E-AAC/M- CTT	31	22	71.01	55–220	0.45	0.41
E-ACT/M-CTG	49	31	63.27	50–560	0.41	0.36
E-ACA/M-CAG	42	21	50.00	95–520	0.32	0.29
E-ACT/M-CAG	55	51	92.73	75–490	0.48	0.44
E-AGG/M-CTA	69	52	75.36	90–785	0.35	0.32
E-AAG/M-CTT	31	18	58.06	95–610	0.44	0.41
E-ACT/M-CTT	62	43	69.36	50–520	0.42	0.38
E-ACC/M-CTA	44	34	77.27	60–540	0.45	0.42
E-AGG/M-CAG	28	19	67.86	55–490	0.39	0.36
E-AAG/M-CTG	72	41	56.94	45–540	0.17	0.14
E-AGG/M-CAC	68	51	75.00	60–560	0.44	0.39
E-ACC/M-CAC	73	37	50.68	55–750	0.22	0.19
E-AGG/M-CTT	61	44	72.13	65–490	0.43	0.39
E-ACC/M-CAG	75	34	45.33	75–780	0.19	0.15
Mean	54.29	35.57	66.07	-	0.37	0.33

*M*, 5′-GATGAGTCCTGAGTAA-3′; *E*, 5′-GACTGCGTACCAATTC-3′; *PIC*, polymorphic information content.

**Table 2:** Genetic variability indices of the 40 barley accessions analyzed.

Accession Number	Cultivar Name	Origin	*H*_o_	*H*_e_	*F*	*PIC*
CGN00328	Albert	France	0.312	0.415	0.301	0.381
CGN02709	Ares	France	0.325	0.391	0.214	0.362
CGN02712	Astrix	France	0.213	0.289	0.211	0.244
CGN00329	Baronne	France	0.332	0.428	0.282	0.392
CGN11185	Beatrice	France	0.274	0.337	0.182	0.298
CGN00350	Berenice	France	0.261	0.368	0.298	0.331
CGN00330	Berrichonne	France	0.318	0.411	0.305	0.379
CGN00318	Betina	France	0.217	0.299	0.245	0.262
CGN00351	Carmen	France	0.131	0.126	0.041	0.103
CGN00339	Ceres	France	0.253	0.326	0.212	0.291
CGN00337	Chevalier	France	0.127	0.226	0.303	0.203
CGN00325	Comtesse	France	0.247	0.357	0.332	0.317
CGN21737	Cytris	France	0.317	0.431	0.368	0.391
CGN02043	Damseaux	France	0.286	0.469	−0.537	0.481
CGN16158	Flamenco	France	0.216	0.325	0.336	0.292
CGN23651	Gerbel	France	0.292	0.427	0.392	0.391
CGN00008	Agio	Netherlands	0.263	0.352	0.274	0.312
CGN19325	Anoa	Netherlands	0.165	0.257	0.278	0.223
CGN21734	Apex	Netherlands	0.262	0.324	0.151	0.281
CGN21735	Bellona	Netherlands	0.236	0.326	0.274	0.285
CGN00003	Bigo	Netherlands	0.142	0.206	0.246	0.104
CGN00016	Cambrinus	Netherlands	0.221	0.207	0.031	0.106
CGN19319	Cumbia	Netherlands	0.142	0.235	0.314	0.192
CGN00014	Delta	Netherlands	0.253	0.341	0.274	0.298
CGN19330	Efron	Netherlands	0.267	0.363	0.302	0.325
CGN00230	Germania	Netherlands	0.273	0.327	0.151	0.284
CGN19326	Grosso	Netherlands	0.287	0.364	0.241	0.328
CGN08532	Hexa	Netherlands	0.291	0.425	0.361	0.385
CGN00450	Antalek	Poland	0.353	0.442	0.271	0.478
CGN00449	Damazy	Poland	0.226	0.389	0.392	0.449
CGN00385	Danzig	Poland	0.253	0.432	0.461	0.398
CGN00407	Groesjetski	Poland	0.297	0.454	0.411	0.413
CGN02119	Kujawiak 2	Poland	0.184	0.306	0.352	0.299
CGN02106	Kujawiak	Poland	0.288	0.437	0.373	0.391
CGN00451	Lubuski	Poland	0.198	0.328	0.342	0.284
CGN00448	Skreszowicki	Poland	0.349	0.242	0.031	0.311
CGN02107	Slaski 1	Poland	0.185	0.278	0.272	0.279
CGN02109	Slaski 2	Poland	0.374	0.501	−0.393	0.482
CGN02118	Urbanowicki	Poland	0.311	0.463	−0.411	0.480
CGN02103	W.R.112 Grodkowicki	Poland	0.187	0.278	0.273	0.233
Mean			0.253	0.348	0.281	0.311

*H*_o_, observed heterozygosity; *F*, fixation index; *H*_e_, expected heterozygosity; *PIC*, polymorphic information content.

**Table 3:** Genetic variability indices of barley accessions in the two European regions.

Barley Group	*H*_o_	*H*_e_	*F*	*PIC*
Western Europe	0.247	0.335	0.261	0.298
Eastern Europe	0.267	0.380	0.327	0.368

*H*_o_, observed heterozygosity; *F*, fixation index; *H*_e_, expected heterozygosity; *PIC*, polymorphic. information content.

**Table 4:** Contents of chlorophyll, carotenoids, proteins, sugars, proline, phenolics, flavonoids, H_2_O_2_, malondialdehyde (MDA) and antioxidant activity (DPPH) in leaves of Slaski 2 cultivar under salt and/or ALA (7 ppm) treatments.

NaCl Treatment	0 mM		100 mM	
	−ALA	+ALA	−ALA	+ALA
Chlorophyll a (mg·g^−1^ FW)	2.71 ± 0.13 ^c^	2.98 ± 0.15 ^a^	2.59 ± 0.11 ^c^	2.80 ± 0.13 ^b^
Chlorophyll b (mg·g^−1^ FW)	1.39 ± 0.06 ^c^	1.71 ± 0.06 ^a^	1.33 ± 0.04 ^c^	1.51 ± 0.07 ^b^
Total Chl (mg·g^−1^ FW)	4.10 ± 0.12 ^c^	4.69 ± 0.14 ^a^	3.99 ± 0.17 ^c^	4.31 ± 0.14 ^b^
Carotenoid (mg·g^−1^ FW)	0.32 ± 0.06 ^c^	0.37 ± 0.07 ^a^	0.30 ± 0.09 ^c^	0.34 ± 0.06 ^b^
Proteins (mg·g^−1^ FW)	0.92 ± 0.11 ^c^	1.07 ± 0.07 ^a^	0.86 ± 0.08 ^c^	0.98 ± 0.09 ^b^
Soluble sugars (µg·g^−1^ FW)	2.12 ± 0.16 ^c^	2.35 ± 0.12 ^a^	2.04 ± 0.14 ^c^	2.19 ± 0.17 ^b^
Proline (µg·g^−1^ FW)	14.4 ± 0.47 ^d^	21.8 ± 0.51 ^c^	25.9 ± 0.42 ^b^	30.8 ± 0.51 ^a^
Total Phenolics (µmol·g^−1^ FW)	14.3 ± 0.33 ^c^	16.8 ± 0.41 ^a^	13.1 ± 0.31 ^c^	14.8 ± 0.32 ^b^
Total Flavonoids (µmol·g^−1^ FW)	7.11 ± 0.18 ^c^	8.89 ± 0.15 ^a^	6.41 ± 0.17 ^c^	7.22 ± 0.13 ^b^
H_2_O_2_ (µmol·g^−1^ FW)	0.23 ± 0.03 ^c^	0.18 ± 0.05 ^d^	0.34 ± 0.04 ^a^	0.29 ± 0.06 ^b^
MDA (µmol·g^−1^ FW)	0.17 ± 0.05 ^c^	0.15 ± 0.04 ^d^	0.28 ± 0.03 ^a^	0.23 ± 0.05 ^b^
DPPH (IC_50_, μg·mL^−1^)	0.35 ± 0.02 ^a^	0.32 ± 0.04 ^c^	0.36 ± 0.02 ^b^	0.31 ± 0.02 ^d^

Different alphabetical letters presented in the same row refer to significant differences among the applied treatments (*p* ≤ 0.05).

**Table 5:** Contents of chlorophylls, carotenoids, proteins, sugars, proline, phenolics, flavonoids, H_2_O_2_, MDA and antioxidant activity (DPPH) in leaves of Damseaux cultivar under salt and/or ALA (7 ppm) treatments.

NaCl Treatment	0 mM		100 mM	
	−ALA	+ALA	−ALA	+ALA
Chlorophyll a (mg·g^−1^ FW)	2.67 ± 0.14 ^c^	2.93 ± 0.13 ^a^	2.59 ± 0.13 ^c^	2.81 ± 0.14 ^b^
Chlorophyll b (mg·g^−1^ FW)	1.41 ± 0.07 ^c^	1.69 ± 0.05 ^a^	1.33 ± 0.06 ^c^	1.48 ± 0.05 ^b^
Total Chl (mg·g^−1^ FW)	4.08 ± 0.14 ^c^	4.62 ± 0.11 ^a^	3.98 ± 0.13 ^c^	4.29 ± 0.13 ^b^
Carotenoid (mg·g^−1^ FW)	0.32 ± 0.05 ^c^	0.41 ± 0.04 ^a^	0.29 ± 0.07 ^c^	0.35 ± 0.04 ^b^
Proteins (mg·g^−1^ FW)	0.86 ± 0.08 ^c^	1.11 ± 0.09 ^a^	0.82 ± 0.07 ^c^	1.03 ± 0.07 ^b^
Soluble sugars (µg·g^−1^ FW)	2.07 ± 0.12 ^c^	2.27 ± 0.11 ^a^	2.02 ± 0.15 ^c^	2.17 ± 0.13 ^b^
Proline (µg·g^−1^ FW)	14.1 ± 0.39 ^d^	19.2 ± 0.41 ^c^	24.3 ± 0.37 ^b^	32.2 ± 0.43 ^a^
Total Phenolics (µmol·g^−1^ FW)	13.6 ± 0.22 ^c^	14.9 ± 0.27 ^a^	12.9 ± 0.29 ^c^	14.1 ± 0.27 ^b^
Total Flavonoids (µmol·g^−1^ FW)	6.57 ± 0.13 ^c^	7.97 ± 0.11 ^a^	5.98 ± 0.15 ^c^	6.99 ± 0.12 ^b^
H_2_O_2_ (µmol·g^−1^ FW)	0.21 ± 0.05 ^c^	0.17 ± 0.04 ^d^	0.31 ± 0.06 ^a^	0.26 ± 0.07 ^b^
MDA (µmol·g^−1^ FW)	0.15 ± 0.03 ^c^	0.13 ± 0.05 ^d^	0.26 ± 0.06 ^a^	0.21 ± 0.05 ^b^
DPPH (IC_50_, μg·mL^−1^)	0.33 ± 0.03 ^a^	0.31 ± 0.05 ^c^	0.34 ± 0.03 ^b^	0.29 ± 0.04 ^d^

Different alphabetical letters in the same row show significant differences among the applied treatments (*p* ≤ 0.05).

**Table 6:** Contents of chlorophyll, carotenoids, proteins, sugars, proline, phenolics, flavonoids, H_2_O_2_, MDA and antioxidant activity (DPPH) in leaves of Urbanowicki cultivar under salt and/or ALA (7 ppm) treatments.

NaCl Treatment	0 mM		100 mM	
	−ALA	+ALA	−ALA	+ALA
Chlorophyll a (mg·g^−1^ FW)	2.69 ± 0.11 ^c^	2.88 ± 0.15 ^a^	2.57 ± 0.12 ^c^	2.74 ± 0.13 ^b^
Chlorophyll b (mg·g^−1^ FW)	1.39 ± 0.04 ^c^	1.58 ± 0.06 ^a^	1.32 ± 0.05 ^c^	1.41 ± 0.04 ^b^
Total Chl (mg·g^−1^ FW)	4.08 ± 0.13 ^c^	4.46 ± 0.14 ^a^	3.96 ± 0.12 ^c^	4.15 ± 0.11 ^b^
Carotenoid (mg·g^−1^ FW)	0.31 ± 0.06 ^c^	0.38 ± 0.05 ^a^	0.29 ± 0.06 ^c^	0.37 ± 0.07 ^b^
Proteins (mg·g^−1^ FW)	0.91 ± 0.07 ^c^	1.22 ± 0.08 ^a^	0.85 ± 0.08 ^c^	1.07 ± 0.06 ^b^
Soluble sugars (µg·g^−1^ FW)	2.03 ± 0.11 ^c^	2.18 ± 0.13 ^a^	1.98 ± 0.12 ^c^	2.08 ± 0.11 ^b^
Proline (µg·g^−1^ FW)	13.9 ± 0.29 ^d^	18.3 ± 0.38 ^c^	25.7 ± 0.27 ^b^	31.8 ± 0.34 ^a^
Total Phenolics (µmol·g^−1^ FW)	13.8 ± 0.21 ^c^	14.6 ± 0.22 ^a^	12.9 ± 0.25 ^c^	14.1 ± 0.23 ^b^
Total Flavonoids (µmol·g^−1^ FW)	7.05 ± 0.13 ^c^	7.86 ± 0.12 ^a^	5.98 ± 0.11 ^c^	7.02 ± 0.13 ^b^
H_2_O_2_ (µmol·g^−1^ FW)	0.19 ± 0.06 ^c^	0.16 ± 0.03 ^d^	0.28 ± 0.08 ^a^	0.23 ± 0.05 ^b^
MDA (µmol·g^−1^ FW)	0.14 ± 0.05 ^c^	0.11 ± 0.03 ^d^	0.24 ± 0.07 ^a^	0.19 ± 0.06 ^b^
DPPH (IC_50_, μg·mL^−1^)	0.31 ± 0.04 ^a^	0.29 ± 0.04 ^b^	0.32 ± 0.02 ^c^	0.30 ± 0.03 ^d^

Different alphabetical letters in the same row show significant differences among the applied treatments (*p* ≤ 0.05).

**Table 7:** Contents of chlorophyll, carotenoids, proteins, sugars, proline, phenolics, flavonoids, H_2_O_2_, MDA and antioxidant activity (DPPH) in leaves of Carmen cultivar under salt and/or ALA (7 ppm) treatments.

NaCl Treatment	0 mM		100 mM	
	−ALA	+ALA	−ALA	+ALA
Chlorophyll a (mg·g^−1^ FW)	2.44 ± 0.11 ^b^	2.86 ± 0.13 ^a^	1.78 ± 0.16 ^d^	2.11 ± 0.12 ^c^
Chlorophyll b (mg·g^−1^ FW)	1.21 ± 0.08 ^b^	1.68 ± 0.07 ^a^	0.92 ± 0.05 ^d^	1.18 ± 0.08 ^c^
Total Chl (mg·g^−1^ FW)	3.65 ± 0.13 ^b^	4.54 ± 0.11 ^a^	2.70 ± 0.14 ^d^	3.29 ± 0.10 ^c^
Carotenoid (mg·g^−1^ FW)	0.29 ± 0.04 ^b^	0.34 ± 0.08 ^a^	0.22 ± 0.03 ^c^	0.28 ± 0.04 ^b^
Proteins (mg·g^−1^ FW)	0.89 ± 0.08 ^b^	1.03 ± 0.09 ^a^	0.78 ± 0.11 ^d^	0.85 ± 0.12 ^c^
Soluble sugars (µg·g^−1^ FW)	2.04 ± 0.13 ^b^	2.19 ± 0.14 ^a^	1.87 ± 0.12 ^d^	1.96 ± 0.14 ^c^
Proline (µg·g^−1^ FW)	14.8 ± 0.38 ^d^	22.7 ± 0.44 ^c^	26.7 ± 0.38 ^b^	32.6 ± 0.46 ^a^
Total Phenolics (µmol·g^−1^ FW)	13.9 ± 0.36 ^b^	15.2 ± 0.32 ^a^	09.1 ± 0.29 ^d^	11.8 ± 0.31 ^c^
Total Flavonoids (µmol·g^−1^ FW)	6.23 ± 0.13 ^b^	8.25 ± 0.16 ^a^	4.14 ± 0.14 ^d^	5.07 ± 0.16 ^c^
H_2_O_2_ (µmol·g^−1^ FW)	0.25 ± 0.03 ^c^	0.21 ± 0.06 ^d^	0.68 ± 0.05 ^a^	0.51 ± 0.04 ^b^
MDA (µmol·g^−1^ FW)	0.19 ± 0.04 ^c^	0.17 ± 0.03 ^d^	0.51 ± 0.05 ^a^	0.32 ± 0.04 ^b^
DPPH (IC_50_, μg·mL^−1^)	0.36 ± 0.04 ^a^	0.34 ± 0.03 ^b^	0.33 ± 0.04 ^c^	0.32 ± 0.02 ^d^

Different alphabetical letters in the same row exhibit significant differences among the applied treatments (*p* ≤ 0.05).

**Table 8:** Contents of chlorophyll, carotenoids, proteins, sugars, proline, phenolics, flavonoids, H_2_O_2_, MDA and antioxidant activity (DPPH) in leaves of Bigo cultivar under salt and/or ALA (7 ppm) treatments.

NaCl Treatment	0 mM		100 mM	
	−ALA	+ALA	−ALA	+ALA
Chlorophyll a (mg·g^−1^ FW)	2.47 ± 0.12 ^b^	2.68 ± 0.17 ^a^	1.81 ± 0.14 ^d^	2.23 ± 0.15 ^c^
Chlorophyll b (mg·g^−1^ FW)	1.18 ± 0.04 ^b^	1.44 ± 0.07 ^a^	0.88 ± 0.06 ^c^	1.19 ± 0.06 ^b^
Total Chl (mg·g^−1^ FW)	3.65 ± 0.15 ^b^	4.12 ± 0.12 ^a^	2.69 ± 0.11 ^d^	3.42 ± 0.12 ^c^
Carotenoid (mg·g^−1^ FW)	0.27 ± 0.05 ^b^	0.32 ± 0.04 ^a^	0.18 ± 0.06 ^d^	0.24 ± 0.05 ^c^
Proteins (mg·g^−1^ FW)	0.82 ± 0.07 ^b^	1.08 ± 0.09 ^a^	0.61 ± 0.12 ^c^	0.83 ± 0.11 ^b^
Soluble sugars (µg·g^−1^ FW)	2.01 ± 0.11 ^b^	2.23 ± 0.11 ^a^	1.81 ± 0.13 ^d^	1.93 ± 0.11 ^c^
Proline (µg·g^−1^ FW)	14.1 ± 0.31 ^d^	21.2 ± 0.37 ^c^	27.5 ± 0.41 ^b^	31.3 ± 0.27 ^a^
Total Phenolics (µmol·g^−1^ FW)	12.8 ± 0.41 ^b^	15.7 ± 0.27 ^a^	08.3 ± 0.32 ^d^	12.1 ± 0.33 ^c^
Total Flavonoids (µmol·g^−1^ FW)	6.11 ± 0.11 ^b^	8.12 ± 0.12 ^a^	4.03 ± 0.16 ^d^	5.22 ± 0.15 ^c^
H_2_O_2_ (µmol·g^−1^ FW)	0.23 ± 0.05 ^c^	0.20 ± 0.05 ^d^	0.71 ± 0.06 ^a^	0.57 ± 0.07 ^b^
MDA (µmol·g^−1^ FW)	0.18 ± 0.06 ^c^	0.15 ± 0.06 ^d^	0.59 ± 0.08 ^a^	0.39 ± 0.06 ^b^
DPPH (IC_50_, μg·mL^−1^)	0.34 ± 0.03 ^a^	0.32 ± 0.04 ^b^	0.31 ± 0.03 ^c^	0.29 ± 0.03 ^d^

Different alphabetical letters in the same row show significant differences among the applied treatments (*p* ≤ 0.05).

**Table 9:** Contents of chlorophyll, carotenoids, proteins, sugars, proline, phenolics, flavonoids, H_2_O_2_, MDA and antioxidant activity (DPPH) in leaves of Cambrinus cultivar under salt and/or ALA (7 ppm) treatments.

NaCl Treatment	0 mM		100 mM	
	−ALA	+ALA	−ALA	+ALA
Chlorophyll a (mg·g^−1^ FW)	2.39 ± 0.12 ^b^	2.72 ± 0.15 ^a^	1.71 ± 0.13 ^d^	2.22 ± 0.11 ^c^
Chlorophyll b (mg·g^−1^ FW)	1.28 ± 0.05 ^b^	1.56 ± 0.08 ^a^	0.91 ± 0.04 ^d^	1.21 ± 0.07 ^c^
Total Chl (mg·g^−1^ FW)	3.67 ± 0.12 ^b^	4.28 ± 0.11 ^a^	2.62 ± 0.13 ^d^	3.43 ± 0.12 ^c^
Carotenoid (mg·g^−1^ FW)	0.27 ± 0.05 ^b^	0.36 ± 0.06 ^d^	0.20 ± 0.04 ^c^	0.25 ± 0.05 ^c^
Proteins (mg·g^−1^ FW)	0.86 ± 0.07 ^b^	1.15 ± 0.07 ^a^	0.71 ± 0.12 ^d^	0.82 ± 0.13 ^c^
Soluble sugars (µg·g^−1^ FW)	1.98 ± 0.11 ^c^	2.21 ± 0.12 ^a^	1.81 ± 0.11 ^d^	2.06 ± 0.12 ^b^
Proline (µg·g^−1^ FW)	16.1 ± 0.31 ^d^	24.2 ± 0.33 ^c^	27.2 ± 0.34 ^b^	33.4 ± 0.38 ^a^
Total Phenolics (µmol·g^−1^ FW)	14.1 ± 0.34 ^b^	15.6 ± 0.35 ^a^	10.2 ± 0.37 ^d^	13.7 ± 0.33 ^c^
Total Flavonoids (µmol·g^−1^ FW)	6.88 ± 0.12 ^b^	8.11 ± 0.14 ^a^	4.03 ± 0.12 ^d^	5.04 ± 0.13 ^c^
H_2_O_2_ (µmol·g^−1^ FW)	0.23 ± 0.07 ^c^	0.20 ± 0.05 ^d^	0.72 ± 0.07 ^a^	0.55 ± 0.06 ^b^
MDA (µmol·g^−1^ FW)	0.18 ± 0.05 ^c^	0.15 ± 0.04 ^d^	0.56 ± 0.04 ^a^	0.38 ± 0.03 ^b^
DPPH (IC_50_, μg·mL^−1^)	0.35 ± 0.02 ^a^	0.33 ± 0.03 ^b^	0.32 ± 0.02 ^c^	0.31 ± 0.02 ^d^

Different alphabetical letters in the same row show significant differences among the applied treatments (*p* ≤ 0.05).
